# The incidence and clinical characteristics of fragile X syndrome in China

**DOI:** 10.3389/fped.2023.1064104

**Published:** 2023-02-13

**Authors:** Lianni Mei, Chunchun Hu, Dongyun Li, Ya Wang, Huiping Li, Kaifeng Zhang, Bingrui Zhou, Ruoping Zhu, Randi J. Hagerman, Xiu Xu, Qiong Xu

**Affiliations:** ^1^ Department of Child Health Care, Children's Hospital of Fudan University, Shanghai, China; ^2^ Department of Child Health Care, Anhui Provincial Children's Hospital, Hefei, China; ^3^ The MIND Institute, University of California Davis Medical Center, Sacramento, CA, United States; ^4^ Department of Pediatrics, University of California Davis Medical Center, Sacramento, CA, United States

**Keywords:** FXS, incidence, clinical characteristics, NDD, children

## Abstract

**Introduction:**

Fragile X syndrome (FXS) is a X-linked neurodevelopmental disorder (NDD). This study aims to investigate the incidence of FXS in Chinese children and analyze the comprehensive clinical characteristics of these FXS children.

**Methods:**

Children diagnosed with idiopathic NDD were recruited between 2016 and 2021 from the department of Child Health Care, Children's Hospital of Fudan University. We combined tetraplet-primed PCR-capillary electrophoresis and whole exome sequencing (WES)/panel or array-based comparative genomic hybridization (array-CGH) to identify the size of the CGG repeats and the mutations or copy number variations (CNVs) in the genome and in *FMR1*. The clinical features of FXS children were analyzed according to pediatricians' recording, parental questionnaires, the results of examinations and follow-up.

**Results:**

The incidence of FXS in Chinese children with idiopathic NDD was 2.4% (42/1753) and in those with FXS, 2.38% had a deletion (1/42). Here, we present the clinical characteristics of 36 children with FXS. Overweight was observed in two boys. The average intelligence quotient (IQ)/development quotient (DQ) of all FXS patients was 48. The average ages of meaningful words and walking alone were 2 years and 10 months and 1 year and 7 months, respectively. The most frequent repetitive behavior was stimulated by hyperarousal to sensory stimulation. On social aspects, social withdrawal, social anxiety, and shyness accounted for 75%, 58%, and 56% of the total number of children, respectively. Approximately 60% of FXS children in this cohort were emotionally labile and prone to temper tantrums. Self-injury and aggression toward others could also be observed, at 19% and 28%, respectively. The most frequent behavioral problem was attention-deficit hyperactivity disorder (ADHD) seen in 64% and the most common facial features were a narrow and elongated face and large or prominent ears in 92% of patients.

**Discussion:**

Screening of *FMR1* full mutation provides the possibility for patients' further medical supports and the clinical features of FXS children obtained in this study will increase the understanding and diagnosis of FXS.

## 1. Introduction

Fragile X syndrome (FXS) (#OMIM300624) is an X-linked neurodevelopmental disorder (NDD) that is the most common inherited cause of intellectual disability (ID) and the most prevalent monogenic cause of autism spectrum disorder (ASD) (1). FXS is mostly caused by an expansion of CGG trinucleotide repeats over 200 in the 5′untranslated region of the fragile X messenger ribonucleoprotein 1 gene (*FMR1*, OMIM309550), located on Xq27.3. The abnormal CGG expansion results in a methylated silencing of the *FMR1* gene and a reduction or absence of mRNA and the encoded protein, FMRP (2). The reduction or loss of FMRP in a small number of FXS patients is caused by point mutations or deletion of *FMR1* (3). FMRP is an mRNA binding protein and a translational regulator. FMRP inhibits the translation of numerous genes involved in synaptic development and plasticity, which leads to abnormalities in neurodevelopmental processes and deficits in learning and memory (4, 5). There are four *FMR1* expanded types based on the different length of CGG repeats. CGG repeats between 5 and 44 are normal, between 45 and 54 CGG repeats are considered a “gray zone,” between 55 and 200 CGG repeats are called premutation (PM), and CGG repeats greater than 200 are defined as full mutation (FM). The *FMR1* premutation has been linked to other disorders such as a late-onset neurodegenerative syndrome, fragile X-associated tremor/ataxia syndrome (FXTAS), fragile X-associated neuropsychiatric disorder (FXAND), and fragile X-associated primary ovarian insufficiency (FXPOI) (6).

It has been estimated that FXS in the general population affects approximately 1 in 5,000 males and 1 in 4,000–8,000 females (7). In Western countries, two large-scale population studies have been carried out in neonates. One study found seven FXS males after screening 36,124 newborn males in Georgia, United States, and revealed the prevalence of full mutation in males of 1 in 5,161 (8). Another study found two FXS males after screening 24,449 neonates in Québec, Canada, and revealed the prevalence of full mutation in males of 1 in 6,209 (9). In China, there was large-scale screening for *FMR1* allele frequencies in 51,000 newborns (28,114 males and 23,547 females) in Hangzhou, Zhejiang, which revealed that the frequency of CGG repeats over 100 was 1/9,371 in males and 1/5,887 in females. However, this article did not report the newborn numbers of CGG repeats over 200 (10). Peprah summarized the incidence of FXS in the ID population of different countries, which ranged from 0.5% to 9.7% (11). In the past, a small number of studies investigated the incidence of FXS in the ID population in China. Chen et al. and Pang et al. reported that the incidence of FXS in Chinese children with unknown ID was 0.93% (5/540) and 0.6% (2/324), which were lower than that reported from studies in Western counties (12, 13). Zhong et al. and Shen et al., respectively, found that 2.8% (32/1127) and 6.8% (6/88) of the Chinese ID population screened by DNA analysis had full mutation. The incidence was not apparently different from that of Caucasian subjects, which ranged from 2.6 to 8.7% (14, 15). Another study using a small sample size reported that the incidence of full mutation was 21.2% (7/33) in children with developmental delay, suggesting that the incidence was higher than that previously reported (10). From these, it seems clear that there is relatively little published data on FXS screening and the results are quite different in China, but it is likely to vary depending on the region. Therefore, it is of urgent necessity to further carry out large-scale FXS screening in the Chinese population.

The typical clinical characteristics of FXS in males are mild to severe ID, language delay, behavioral problems such as autism, hyperactivity, short attention span, anxiety, well-known facial features including a narrow and elongated face, a broad forehead, large or prominent ears, and a prominent jaw. Some have comorbid problems such as epilepsy, gastroesophageal reflux, mitral valve prolapse, and scoliosis (16). Females with FXS are less affected than males with FXS because of the second X chromosome that produces FMRP depending on the activation ratio (17). However, the clinical features evolve with age and the facial features are more prominent with age, whereas the hyperextensible finger joints are less prominent with age. Those with size mosaicism have less obvious physical features because they are producing more FMRP. No previous study has investigated the comprehensive FXS clinical characteristics of Chinese children, and this study aims to fill this gap and will lead to a better identification of FXS in China.

This study investigated the incidence of FXS in children with idiopathic NDD admitted to The Children's Hospital of Fudan University from 2016 to 2021 and analyzed the comprehensive clinical characteristics of these children; it aims to provide a basis for precise diagnosis and targeted treatments.

## 2. Methods

### 2.1. Subjects

A total of 1,753 children diagnosed with idiopathic neurodevelopmental disorder were recruited between 2016 and 2021 from the department of Child Health Care, Children's Hospital of Fudan University. Informed consent was obtained from patients or their guardians for genetic testing and further clinical evaluations. The recruited patients met at least one of the first two requirements and the third requirement:

– Patients with unknown global developmental disorder (GDD) or ID. GDD diagnosis was based on DQ lower than 70 in two or more developmental domains of Griffiths development scale—Chinese. ID diagnosis was based on IQ lower than 70 of Wechsler Preschool and Primary Scale of Intelligence (WPPSI) or Wechsler Intelligence Scale for Children (WISC).
– Patients with ASD including DQ/IQ above 70. ASD diagnosis was based on the Diagnostic and Statistical Manual of Mental Disorders, fifth edition (DSM-V) criteria.
– Patients did not have craniocerebral trauma, limb malformation, or severe multiple organ malformation.

### 2.2. Genetic studies

**Figure 1:**
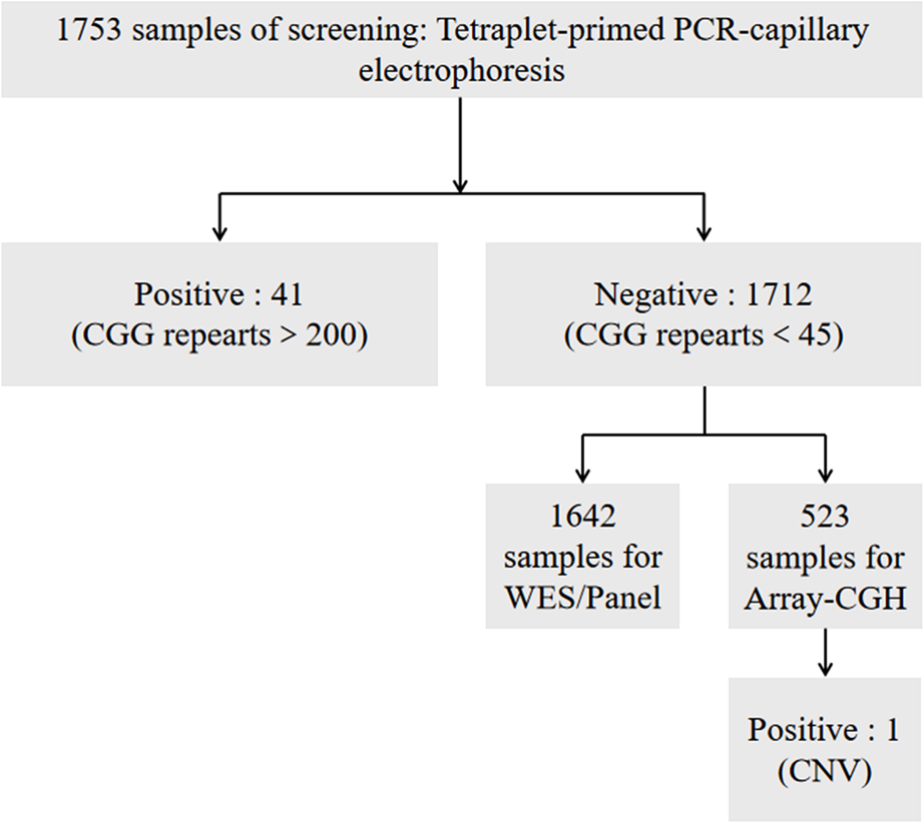
The procedure of FXS screening.

The procedure of the FXS screening is illustrated in Figure 1, which has two steps. First step: Genomic DNA was extracted from peripheral blood samples in EDTA-coated Vacutainers. The 1,753 samples were first detected by using *FMR1* CGG repeat PCR and processed for the capillary electrophoresis (CE) analysis. All samples were prepared for an laboratory developed test (LDT) FXS screening PCR with Roche FastStart™ Taq and a master mix containing a 10X PCR reaction buffer and 2.5 mM dNTP, a 10 mM 7-deaza-dGTP, a 25 mM MgCl2, and a GC-rich solution. The primer mix contained an fluorescein amidites (FAM)-labeled forward, reverse primer of FMR1 5′UTR and an FMR1 CGG-specific primer. A quantity of 1 μl of gDNA was transferred to the PCR master mix to amplify with an initial denaturation step of 95 °C for 5 min, 10 cycles of 97 °C for 35 s, 62 °C for 35 s, and 68 °C for 4 min, then 20 cycles of 97 °C for 35 s, 62 °C for 35 s, and 68 °C for 4 min with a 20-s extension at each cycle, and a final extension at 72 °C for 10 min. The ABI 3,130 Genetic Analyzer (Applied Biosystems, Foster City, CA, United States) was applied for *FMR1* CGG repeat fragment analysis. The 0.1 μl of PCR product was transferred into a 96-well plate with 1% 500 LIZ™ Size Standard (Applied Biosystems, Foster City, CA, United States) in 10 μl Hi-Di™ Formamide (Applied Biosystems, Foster City, CA, United States). After capillary electrophoresis, the fragment analysis data interpretation was performed by using GeneMarker® (SoftGenetics, PA, United States).

Second step: A majority of the negative samples were further tested by using (WES)/panel and (array-CGH) to confirm whether there were mutations or CNVs in the genome and in *FMR1*.

### 2.3. Clinical evaluations

To elucidate the clinical characteristics of Chinese FXS children, we analyzed all their medical history. Clinical data mainly included the following:

– Behavioral problems and family history were assessed and recorded by pediatrician observation and parental questionnaires.
– Physical examinations including general growth and development were performed by pediatricians during the clinical visit. Each child with FXS received face photography.
– Relevant examinations such as electrocardiogram, echocardiogram, electroencephalogram, and spine x-ray were reviewed.
– Follow-up results of the ophthalmology, otolaryngology, gastroenterology, and neurology departments were documented.

### 2.4. Statistical analysis

Demographic and clinical data were analyzed using the statistical package SPSS 20.0.

## 3. Results

### 3.1. The incidence of FXS in Chinese children with idiopathic NDD

Samples from 1,753 Chinese children with idiopathic NDD (male:female = 1,382:371, age: 0.5–16 years old) were first tested by tetraplet-primed PCR-capillary electrophoresis, and 41 samples with CGG over 200 were detected (male:female = 39:2). Most negative samples were further analyzed for WES/panel or array-CGH. A 5-year-old girl had a 64 kb deletion in Xq27.3, including *FMR1* gene, which was screened by using array-CGH. The incidence of FXS in Chinese children with idiopathic NDD was 2.4%, and those with FXS 2.38% had a deletion (Table 1).

**Table 1:** The incidence of FXS in children with idiopathic NDD.

CGG repeats	Category	Total	Proportion	Male/Female
	>200	42^a^	42/1,753 (2.4%)	39:3
	<200	1,711	1,711/1,753	1,343:368

^a^ One child had a 64 kb deletion in Xq27.3, including the FMR1 gene.

### 3.2. Clinical characteristics of Chinese children with FXS

#### 3.2.1. Basic information, general growth, and development

Clinical data of 42 children with FXS were reviewed, and it was found that the data of 6 children were incomplete. Table 2 shows basic information, general growth, and development related to the 42 FXS children (male:female = 39:3). The mean and median ages at the time of assessment were 4 years old and 4 months, among which the oldest was 9 years old and 11 months, and the youngest was 7 months.

**Table 2:** Statistics of basic information, general growth, and development of FXS children.

Items	Mean (SD)	Median	Maximum and minimum value	Ratio or proportion
Sex	—	—	—	Male: Female = 39:3
Age	7 years 1 month (3 years)	6 years 9 months	13 years 6 months, 1 year 9 months	—
Age at assessment	4 years 4 months (2 years 4 months)	4 years 4 months	9 years 11 months, 7 months	—
Meaningful words	2 years 10 months (1 year 1 month)	3 years	5 years, 1 year 1 months	>1 year 6 months:37/42 (88%)
Walking alone	1 year 7 months (4 months)	1 year 7 months	3 years, 1 year 1 months	>1 year 6 months:20/42 (48%)
Griffiths/WPPSI/WISC	48 (12)	45	69, 28	<70:42/42 (100%)
Physical growth	Type	Total	Male: Female	Proportion
Weight	Overweight	2	2:0	2/42 (5%)

FXS, fragile X syndrome; WPPSI, Wechsler Preschool and Primary Scale of Intelligence; WISC, Wechsler Intelligence Scale for Children.

Growth patterns were mostly within the normal range. However, overweight (according to the curves of the height and body mass index of children developed by the World Health Organization) was also observed in two children. Children's intelligence or development was tested by using WPPSI/WISC or Griffiths. The mean IQ/DQ of all FXS patients was 48 and the median IQ/DQ was 45. We analyzed the two key milestones of the age of meaningful words and walking alone. The mean and median ages at which meaningful words appeared were 2 years and 10 months and 3 years, respectively, and the latest age was 5 years old. A total of 88% of FXS children experienced delay in uttering the first meaningful words. The mean and median ages of walking alone were 1 year and 7 months, and the oldest was 3 years old. A total of 48% of patients experienced delayed walking.

#### 3.2.2. Repetitive behaviors

Table 3 shows the characteristic repetitive behaviors of FXS and the proportion of FXS children with each stereotyped behavior in the total number of patients. The most frequent repetitive behavior was stimulated by hyperarousal to sensory stimulation, accounting for 69%. These sensory stimulations included squinting of the eyes, smelling, covering the ears, touching objects with the mouth or tongue, and turning in circles. Hand-flapping and hypersensitivity for changes were also common in children with FXS, accounting for half of the total. Hand-biting was less frequent, at 25%.

**Table 3:** Proportion of FXS children with certain features in the total number of patients.

Repetitive behaviors	Proportion
Hyperarousal to sensory stimuli	25/36 (69%)
Hand-flapping	18/36 (50%)
Hypersensitivity for changes	18/36 (50%)
Hand-biting	9/36 (25%)
Social and emotional problems	Proportion
Social withdrawal	27/36 (75%)
Social anxiety	21/36 (58%)
Shyness	20/36 (56%)
Tantrums	21/36 (58%)
Mood lability	20/36 (56%)
Aggression	10/36 (28%)
Self-injury	7/36 (19%)
Complication	Proportion
ADHD	23/36 (64%)
Vomiting	12/36 (33%)
Hyperextensible finger joints	11/36 (31%)
Sleeping problems	8/36 (22%)
Strabismus	7/36 (19%)
Flat feet	5/36 (14%)
Epilepsy	3/36 (8%)
Adenoidal hypertrophy	3/36 (8%)
Diarrhea	3/36 (8%)
Constipation	3/36 (8%)
Gastroesophageal reflux	2/36 (6%)
Macroorchidism	1/36 (3%)
Otitis media or urethritis	0
Mitral valve prolapse	0
Scoliosis	0
Facial features	Proportion
Narrow and elongated face	33/36 (92%)
Large or prominent ears	33/36 (92%)
Prominent jaw	25/36 (69%)
Long palpebral fissure	21/36 (58%)
Broad or prominent forehead	19/36 (53%)
Periorbital puffiness	14/36 (39%)
Highly arched palate	0
Crowded teeth or malocclusion	0

#### 3.2.3. Social and emotional problems

Social abnormalities were obvious in children with FXS. Social withdrawal, social anxiety, and shyness accounted for 75%, 58%, and 56% of the total number of children, respectively. In addition, FXS children often suffered from emotional problems. Approximately 60% of FXS children in this cohort were emotionally labile and prone to temper tantrums. Self-injury and aggression toward others could also be observed, accounting for 19% and 28% respectively, and were more common in older children (Table 3).

#### 3.2.4. Complications

Among behavior problems of FXS, the most frequent was ADHD, which was seen in 64% of children. Gastrointestinal symptoms were common in our cohort, with vomiting occurring most frequently in 33%. Others were relatively infrequent: diarrhea and constipation were seen in 8% and gastroesophageal reflux was found in two young children aged 1.5 years old. Hyperextensible finger joints, sleep problems, and strabismus were also common, accounting for 31%, 22%, and 19% respectively. Flat feet were found in five and three FXS children had adenoid hypertrophy. In the total number of patients with FXS, three children had a history of epileptic seizures. A boy aged 6 years and 9 months had two seizures and was treated with oxcarbazepine for 2 years, and a girl aged 2 years and 7 months had one seizure and was treated with oxcarbazepine for 6 months. Another boy aged 5 years and 3 months had one seizure but was not on antiepileptic medication. In our study, only one boy (9 years and 11 months) showed increased testicular volume (25 ml) because the remaining patients were too young for macroorchidism (Table 3).

#### 3.2.5. Facial features

We performed an analysis of children's facial photos because FXS is characterized by craniofacial anomalies. A total of eight facial features were evaluated and all patients had no more than six abnormal features. The most common facial features were a narrow and elongated face and large or prominent ears that presented in 92% of patients, followed by a prominent jaw, a long palpebral fissure, and a broad or prominent forehead (>50%). In addition, we found that approximately 40% of patients had periorbital puffiness (Table 3).

## 4. Discussion

### 4.1. The incidence of FXS in Chinese children with idiopathic NDD

In this study, we determined that the incidence of FXS in Chinese children with idiopathic NDD was 2.4%. This percentage was slightly lower than that reported in Western countries. However, compared with previous studies in China, it was higher than that reported by Chen et al. and Pang et al. (12, 13), 0.93% (5/540) and 0.6% (2/324), respectively, close to that reported by Zhong et al. (14), 2.8% (32/1127), and lower than that reported by Zhang et al. and Shen et al. (10, 15), 21.2% (7/33) and 6.8% (6/88), respectively. Two vital factors can be attributed to the different percentages of incidence of FXS among the Chinese population, and these are the diversity of the recruited populations and different sample sizes of each of these studies. Previous studies conducted FXS screening among people with different degrees of ID and the varying severities of ID among the recruited populations in various studies may affect the results. In this study, FXS screening was carried out in patients with idiopathic NDD and there was an expansion of the recruited populations compared with that in previous studies. In terms of sample size, the percentage of error of research results with small sample sizes may be higher.

We also screened a girl with FXS who had a 64 kb deletion in Xq27.3, including the *FMR1* gene, by using array-CGH. In those with FXS, 2.38% had a deletion. A hyper expansion of CGG trinucleotide repeats over 200 in the 5′untranslated region of the *FMR1* gene is responsible for the majority of FXS cases, and less than 1% of individuals with FXS have a sequence variant, a partial deletion (18). The proportion of CNV, which was more than 1% in our study, may be attributed to the small sample size. On the other hand, this result also indicates the importance of using different research methods to elucidate atypical *FMR1* gene changes.

In recent years, research on FXS-targeted drugs has developed rapidly. Several targeted drugs have been identified, which have the potential to reverse the neurobiological aspects of FXS (19–22). A screening of *FMR1* full mutation provides the possibility for FXS-targeted drug therapy and further medical support.

### 4.2. The clinical characteristics of Chinese FXS children

We elaborated the clinical characteristics of Chinese children with FXS in terms of five factors: general growth and development, repetitive behaviors, social and emotional problems, complications, and facial features.

The height of the children was within the normal range. However, we found that two boys were overweight. The frequency of overweight/obesity was lower than that (53%–61%) previously reported (23). The average IQ/DQ of all FXS patients was 48, which was similar to the mean value of previous reports (23). We showed that the age of meaningful words and walking alone were the two most accurate milestones recorded from the memory of parents. The average age of meaningful words was 2 years and 10 months and that of walking alone was 1 year and 7 months, which were slightly different from those of previous reports (23). The frequency of the age of meaningful words and walking alone older than one and a half years was 88% and 48%, respectively, suggesting that the delay in reaching these two milestones is common in children with FXS.

FXS is the most prevalent monogenic cause of ASD. Individuals with FXS presented several behaviors of autism, such as social abnormalities and repetitive behaviors. Social withdrawal, social anxiety, shyness, hyperarousal to sensory stimulation, and hypersensitivity to changes were frequent in our study (≥50%). Repetitive motor behaviors such as hand-flapping and hand-biting were at 50% and 25%, respectively, which were lower than those (70.8% and 54.2%) reported previously (24). In addition, emotional problems are common in FXS. A total of 56% and 58% of FXS children in this cohort were found to be emotionally labile and prone to temper tantrums. Self-injury accounted for 19%, which was greater than that (15%) in another study (25). Aggressiveness occurred in 28%, which was much lower than that (90%) reported in a previous review (25). This was likely related to cultural differences.

Among complications of FXS, ADHD is considered one of the most common comorbidities of FXS. In our cohort, ADHD accounted for 64%, which was slightly higher than that (54%–59%) reported previously (23). The incidence of vomiting was high at 33% in our cohort. The proportion of other gastrointestinal symptoms was relatively low, and diarrhea and constipation accounted for 8%. Gastroesophageal reflux was found in two young children and the frequency was lower than that (11%) of individuals with FXS in another study (26). Another important complication that occurs in FXS is epilepsy. Other studies have reported the prevalence of epilepsy in FXS individuals at 10%–20% in boys and 5%–10% of girls (23). In our study, 8% of FXS children had epilepsy, and this percentage was similar to that of previous data. Strabismus was seen in 19% of children with FXS, which equaled that of a previous review (26). A large-scale parental survey presented that 32% of FXS children experienced sleeping difficulties, including difficulty in falling asleep and frequent night awakenings (27). Similarly, 22% of FXS children in our study had sleep-related problems. In a previous review, recurrent otitis media was found to be a common medical problem in FXS. However, none of the 36 children had recurrent otitis media in our study, and only three had adenoid hypertrophy with frequent respiratory infections. Problems of connective tissue were also notable in FXS, which may affect the cardiovascular system, limbs, and spine. We found that 11 FXS children had finger joints that were hyperextensible and five had flat feet. However, we did not find any FXS children with mitral valve prolapse, aortic root dilation, or scoliosis after performing an echocardiogram and spinal x-ray. In addition, we found that a boy (9 years and 11 months) had increased testicular volume (25 ml).

In terms of facial features, we found in our study that a narrow and elongated face and large or prominent ears were found in 92% of patients, followed by a prominent jaw (69%). This result was consistent with the typical facial features of FXS reported previously (23). In addition, we found that some facial features were common, such as a long palpebral fissure (58%), a broad or prominent forehead (53%), and periorbital puffiness (39%). Several studies in Japan, Thailand, and Korea reported the facial features of FXS, and some in these countries also found the elongated facial characteristic of FXS to be less significant than that of the American population (28, 29). In sum, the statistics of facial features are subjective to some extent and the facial features become more obvious and noticeable with age.

This is the first FXS clinical study conducted in China to elucidate the clinical characteristics of this syndrome in detail. The clinical features of FXS children obtained in this study will serve to increase the understanding and diagnosis of FXS. These FXS patients' induced pluripotent stem cells (iPSC) derived neurons could be studied in the future as several phenotypic variations were observed compared with published reports. This could lead to a better understanding of the molecular pathogenesis of the disorder.

### 4.3. Limitations of this study

The first limitation of this study is possibly related to the recruitment of children with NDD from the Department of Child Health Care of Children's Hospital of Fudan University in Shanghai, China. This indicates that this is a single-center study, the data of which may be biased because of the lack of a full coverage of the incidence of FXS in NDD in China. The second limitation is that we did not include all 42 children with FXS in the statistical data of clinical phenotypes because of incomplete data, and the total count stood at 36. The sample size of our study is not very large, which may not reflect the facial features of a majority of Chinese children. In addition, clinical data were obtained mainly from pediatricians’ assessments and parental questionnaires, and there is the possibility of these questionnaires being a little subjective in nature. In summary, further studies are needed to continue the exercise of large-scale screening of the *FMR1* gene in the NDD population by involving multiple medical centers in order to provide more accurate epidemiological data. In addition, pediatricians need to master the clinical characteristics of FXS in practice to promote early diagnosis and interventions.

## 5. Conclusions

We determined that the incidence of FXS in Chinese children with idiopathic NDD was 2.4% and the yield of CNV deletions in FXS was 2.38%. In addition, we described the clinical characteristics of Chinese children with FXS from the point of view of five factors: general growth and development, repetitive behaviors, social and emotional problems, complications, and facial features. We found that two children were overweight, and a majority of children experienced delay in the utterance of the first meaningful words and walking alone. All children presented with an abnormal IQ/DQ. Chinese children with FXS presented characteristic behaviors of autism, such as hyperarousal to sensory stimulation, hand-flapping, hand-biting, hypersensitivity to changes, social withdrawal, social anxiety, and shyness. Emotional problems such as mood lability, tantrums, self-injury, and aggressiveness were also common. In terms of complications, ADHD and gastrointestinal symptoms were the most prominent. Epilepsy, strabismus, sleeping problems, and adenoid hypertrophy accounted for a certain proportion, and connective tissue problems such as hyperextensible finger joints and flat feet were seen in some children. However, the well-known macroorchidism of FXS was not found to be significant in our study because the children were mostly prepubertal. A narrow and elongated face (92%), large or prominent ears (92%), and a prominent jaw (69%) were the three most common facial features in this cohort, which were consistent with the typical facial features of FXS reported previously.

## Data Availability

The sequencing data in this paper included WES/panel and array-CGH data. As WES/panel of 1642 samples did not find the mutations in FMR1, it could not be shared due to privacy. From 523 samples tested for array-CGH, only the positive result were available due to privacy. The positive data (1:CNV) has been submitted to dbVar and the accession is nstd226.
