# Corrigendum: The incidence and clinical characteristics of fragile X syndrome in China

**DOI:** 10.3389/fped.2023.1296110

**Published:** 2023-10-18

**Authors:** Lianni Mei, Chunchun Hu, Dongyun Li, Ya Wang, Huiping Li, Kaifeng Zhang, Bingrui Zhou, Ruoping Zhu, Randi J. Hagerman, Xiu Xu, Qiong Xu

**Affiliations:** ^1^ Department of Child Health Care, Children’s Hospital of Fudan University, Shanghai, China; ^2^ Department of Child Health Care, Anhui Provincial Children’s Hospital, Hefei, China; ^3^ The MIND Institute, University of California Davis Medical Center, Sacramento, CA, United States; ^4^ Department of Pediatrics, University of California Davis Medical Center, Sacramento, CA, United States

**Keywords:** FXS, incidence, clinical characteristics, NDD, children

A Corrigendum on The incidence and clinical characteristics of fragile X syndrome in China By Mei L, Hu C, Li D, Wang Y, Li H, Zhang K, Zhou B, Zhu R, Hagerman RJ, Xu X and Xu Q. (2023) Front. Pediatr. 11:1064104. doi: 10.3389/fped.2023.1064104

In the published article, there was no funding statement. The correct Funding statement appears below.

